# Morphology and Molecular Phylogeny of Two Soil Ciliate Species (Protozoa, Ciliophora) from the Changbai Mountain Region, China, Including a New Species

**DOI:** 10.3390/microorganisms14030559

**Published:** 2026-02-28

**Authors:** Yuxuan Wang, Yunhan Wang, Huan Li, Sitong Li, Xuming Pan

**Affiliations:** Key Laboratory of Biodiversity of Aquatic Organisms, Harbin Normal University, Harbin 150025, China

**Keywords:** taxonomy, phylogeny, soil ciliates, morphology, SSU rRNA genes

## Abstract

Soil ciliates are an important component of the soil micro-food web, playing key roles in organic matter decomposition and nutrient cycling. However, research on the species diversity and taxonomy of this group in the temperate forest soils of China is still limited. This study investigates the morphology and ciliary pattern of two ciliate species discovered in the Changbai Mountain region of northeastern China: *Bryometopus changbaishanensis* sp. n. and *Apocolpodidium etoschense* Foissner et al., 2002, using live observation and silver carbonate impregnation. *B*. *changbaishanensis* sp. n. is characterized by the following morphological features: size in vivo approximately 40–48 × 20–29 μm, 11–14 somatic kineties; the paroral membrane consists of about 16–26 dikinetids; and there are 11–15 oral membranelles. This species differs from *B. atypicus* in its smaller body size in vivo, fewer somatic kineties, and fewer oral membranelles. *Apocolpodidium etoschense* Foissner et al., 2002, exhibits the following morphological features: in vivo size approximately 48–85 × 19–35 μm, 16–20 somatic kineties, and a gently curved paroral membrane composed of about 13–20 dikinetids; its hypostomial organelle consists of three to five files, each containing approximately three to five monokinetids. Additionally, DNA extraction and SSU rRNA gene sequencing were performed to elucidate their evolutionary relationships. Phylogenetic analyses based on SSU rRNA gene data indicated that *Bryometopus changbaishanensis* sp. n. clusters with *B. atypicus*. This study also provides a redescription and supplementary definition of *A. etoschense*, with the Changbai Mountain population forming a fully supported cluster with previously sequenced data.

## 1. Introduction

Ciliated protists represent a group of advanced and complex unicellular eukaryotes, characterized by their diverse morphologies and wide distribution across various habitats including marine, freshwater, brackish waters, and soil. They play important ecological roles [1,2,3,4,5,6,7,8,9,10,11,12,13,14,15,16]. Ciliates of the class Colpodea are representative soil-dwelling species, comprising approximately 60 genera and more than 200 species, and they show similar living features, relatively simple body plans and a limited number of morphological characters for species circumscription [17,18]. The genus *Bryometopus*, which belongs to the class Colpodea, is morphologically as follows: it encompasses small to large Bryometopidae with a shallow vestibulum that is parallel or oblique to the longitudinal axis of the cell; the paroral membrane extends along the right margin of the elliptical vestibular trough. The type species, as originally designated, is *B. pseudochilodon* Kahl, 1932 [19]. However, it is noteworthy that most species within this group have not been investigated or reinvestigated using modern methods [18,19,20,21,22].

Ciliates that usually bear a well-developed cytopharynx basket are classified within the class Nassophorea. Foissner et al. established the order Colpodidiida based on the unique stomatogenic pattern and distinct oral region, which includes three oral polykinetids and the cytopharynx supported by a delicate cyrtos, and subsequent phylogenetic analyses confirmed the validity of this assignment [23,24,25,26,27,28]. Lynn [24] acknowledged the aforementioned classifications and summarized the class into four orders, which include Nassulida, Microthoracida, Colpodidiida and Synhymeniida. *Apocolpodidium etoschense* Foissner et al., 2002 belongs to the class Nassophorea [29], order Colpodidiida [27], family Colpodidiidae [30], and genus *Apocolpodidium* [27]. The morphological characteristics of the genus *Apocolpodidium* are as follows: the oral apparatus is comparatively inconspicuous, featuring a small, flat buccal cavity and a short, slightly curved paroral membrane; trichocysts are fusiform; the paroral membrane can be either short and slightly curved or long and C-shaped. The type species is *A. etoschense* Foissner et al., 2002.

This study investigates the morphology and phylogeny of two ciliate species, *Bryometopus changbaishanensis* sp. n. and *Apocolpodidium etoschense* Foissner et al., 2002, collected from soil in the Changbai Mountain region of northeastern China. Additionally, it provides redescriptions and supplementary definitions for *A. etoschense* Foissner et al., 2002. The ciliary pattern is illustrated to clearly demonstrate the morphological characteristics of both ciliates. In addition, molecular phylogenetic analyses based on SSU rRNA sequence data were performed to determine their evolutionary relationships.

## 2. Material and Methods

### 2.1. Sample Collection and Identification

**Figure 1:**
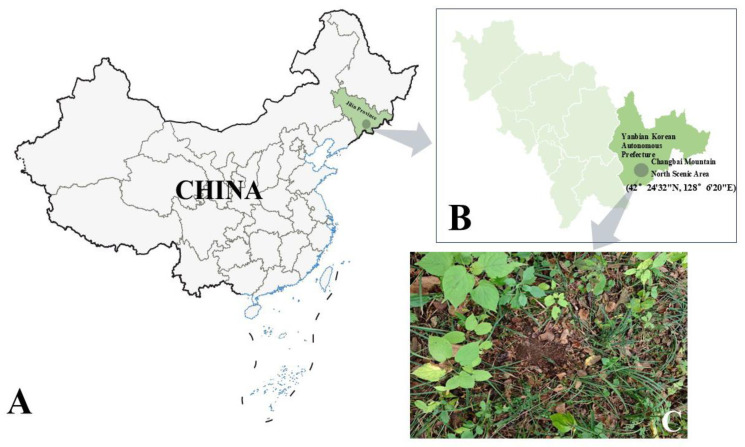
(**A**–**C**) Map and sampling site. (**A**) Map of China, gray circle indicating the location of the sampling site. (**B**) Map of the sampling site in Changbai Mountain. (**C**) Photograph of the sampling site located approximately 2 km southwest of the North Scenic Area of Changbai Mountain (42°24′32″ N, 128°6′20″ E).

*Bryometopus changbaishanensis* sp. n. and *Apocolpodidium etoschense* Foissner et al., 2002 were collected on 17 September 2024 from soil approximately 2 km southwest of the North Scenic Area of Changbai Mountain, near Erdaobaihe Town, Antu County, Yanbian Korean Autonomous Prefecture, Jilin Province, China (42°24′32″ N, 128°6′20″ E). About 0.5 kg of composite dry soil was collected directly from the upper 5 cm of the surface and preserved in a disposable plastic sampling bag without further treatment (Figure 1). The sample was processed with the non-flooded Petri dish method in the laboratory [20]. Ten cells for each species in vivo were observed and photographed using bright field, dark field, and differential interference contrast microscopy (Zeiss, Imager A2). The infraciliature and nuclear apparatus of cells were revealed with the silver carbonate method [31]. Drawings of stained specimens were made using drawing devices (line drawing pens and tracing papers). Measurements were made under 100–1000× magnification. Statistical analyses performed for morphometric data and morphological character selection were according to [24]. Classification and terminology are according to [24,32].

### 2.2. DNA Extraction, PCR Amplification, and Gene Sequencing

For each species, five cells from each monoclone were isolated under the stereomicroscope using micropipettes and washed with double distilled water at least three times to remove contaminants and subsequently incubated in non-nutrient distilled water for 6 h to ensure the removal of any residual food particles. The cells were then transferred into an Eppendorf tube, ensuring the liquid volume did not exceed 5 μL. Total genomic DNA was extracted using the DNeasy & Tissue Kit (DNeasy Blood & Tissue Kit (QIAGEN, Germany, supplied by Shanghai, China) according to the manufacturer’s instructions. The primers EukA 5′-AAC CTG GTT GAT CCT GCC AGT-3′ and EukB 5′-TGA TCC TTC TGC AGG TTC ACC TAC-3′ were used to amplify the SSU rRNA gene [33]. High-fidelity Taq polymerase (Takara Ex Taq; Takara Bio Inc., Otsu, Japan), purchased from Takara Biomedical Technology, Beijing, China) was used to reduce amplification errors. The PCR condition for the SSU-rRNA gene amplification was denaturation for 5 min at 94 °C, followed by 5 cycles of denaturation for 30 s at 94 °C, annealing for 1 min 45 s at 56 °C, extension for 2 min at 72 °C and the other 25 cycles of denaturation for 45 s at 94 °C, annealing for 1 min 45 s at 60 °C, extension for 2 min at 72 °C and a final extension at 72 °C for 8 min [34]. The PCR product purification was performed using the TIANgel Midi Purification Kit (TIANGEN BIOTECH, Beijing, China), cloned using the PMD 18-T vector cloning kit (Takara Biomedicals, Beijing, China), and a randomly selected clone was sequenced bidirectionally in Shanghai Sangon Biological Engineering and Technical Service Company (Shanghai, China).

### 2.3. Phylogenetic Analyses

In this work, 18S rDNA was selected as the gene region in the SSU rRNA gene. Newly generated SSU rRNA gene sequences of *Bryometopus changbaishanensis* sp. n. and *Apocolpodidium etoschense* Foissner et al., 2002 were aligned with the other 62 sequences of ciliates downloaded from the GenBank database. Among these, three sequences of Spirotrichea were selected as the outgroup (for accession numbers, see the respective figure). All the sequences were aligned using Clustal W implemented in BioEdit 7.0.1 [35]. Sequences were aligned using Clustal W 2.1 implemented in BioEdit 7.0 enabling pairwise analysis [35]. The final data set used for phylogenetic analyses comprised 1724 sites.

Phylogenetic trees were inferred using maximum likelihood (ML) and Bayesian inference (BI) methods. ML analyses were constructed by RAxML-HPC2 v8.2.12 [36], and BI analyses by MrBayes v3.2.7a [37], both on the CIPRES Science Gateway. The ML and BI trees based on 18S rRNA gene were constructed according to the GTR + I + G model chosen by the MrModeltest v.2.0 program [38]. ML analysis was performed using rapid bootstrap with 1000 nonparametric bootstrap replicates. Bayesian posterior probabilities were calculated by running four chains for 10,000,000 generations, with the cold chain sampling every 10,000 generations. The remaining trees were used to calculate the posterior probabilities using a majority rule consensus. The average standard deviation of split frequencies (<0.01) was adequate according to [39]. In addition, the results of Estimated Sample Size (ESS) and Potential Scale Reduction Factor (PSRF) were also satisfactory. The first 25% of sampled trees were discarded as burn-in. Support value < 70%/0.94 (ML/BI) was considered as low, 70–95% (ML) as moderate, and >95%/0.95 (ML/BI) as high. MEGA 12.0 [40] was utilized to visualize tree topologies.

## 3. Results

**Class Bryometopia Foissner, 1985.** 

**Order Bryometopida Foissner, 1985.** 

**Family Bryometopidae Jankowski, 1980.** 

**Genus *Bryometopus*** **Kahl, 1932.**

### 3.1. Bryometopus changbaishanensis sp. n.

#### 3.1.1. Diagnosis

The body is about 40–48 μm × 20–29 μm in vivo, and the outline is mostly ovoid, occasionally elongate–ovoid; the contractile vacuole is at the posterior part of cell, with the excretory pore located at its base; there are 11–14 somatic kineties; the buccal cavity is large and deep, and the shallow, droplet-shaped vestibulum leads to the pharynx, occupying approximately 45–50% of the body length; the paroral membrane is composed of about 16–26 dikinetids; the 11–15 oral membranelles are in rows, each comprising two longer rows and one shorter row; the macronucleus and micronucleus are also present in each soil.

#### 3.1.2. Type Locality

The soil was approximately 2 km southwest of the North Scenic Area of Changbai Mountain, near Erdaobaihe Town, Antu County, Yanbian Korean Autonomous Prefecture, Jilin Province, China (42°24′32″ N, 128°6′20″ E).

#### 3.1.3. Type Specimens

The slide containing the holotype specimen (silver nitrate-staining) is deposited in the Laboratory of Protozoology, Harbin Normal University with registration number WYX-2024091701.

#### 3.1.4. Etymology

The species group name ‘*changbaishanensis*’ (Latin adjective; pertaining to Changbai Mountain) refers to the fact that this species was collected from the Changbai Mountain region in Jilin Province, China.

#### 3.1.5. Description

**Figure 2:**
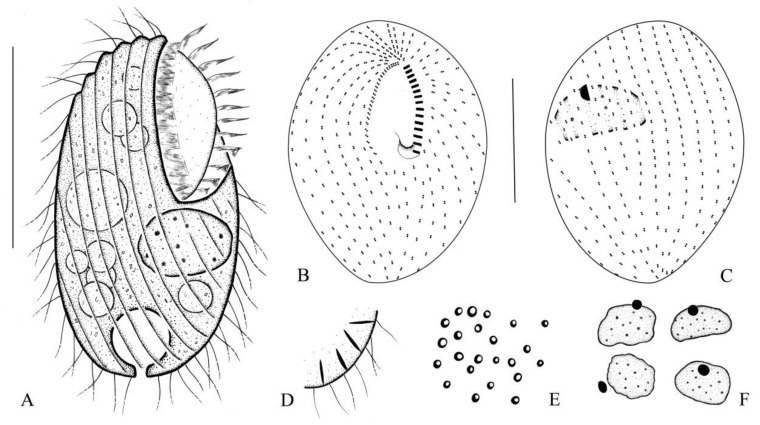
(**A**–**F**) Morphology and ciliary pattern of *Bryometopus changbaishanensis* sp. n. from life (**A**,**D**,**E**) and after silver carbonate staining (**B**,**C**,**F**). (**A**) Typical body shape of a representative specimen. (**B**,**C**) Ventral and dorsal views of infraciliature. (**D**) Extrusomes and somatic cilia. (**E**) Cortical granules in vivo. (**F**) Varied shapes of nuclear apparatus after silver carbonate. Bars, 30 µm (**A**–**C**).

**Figure 3:**
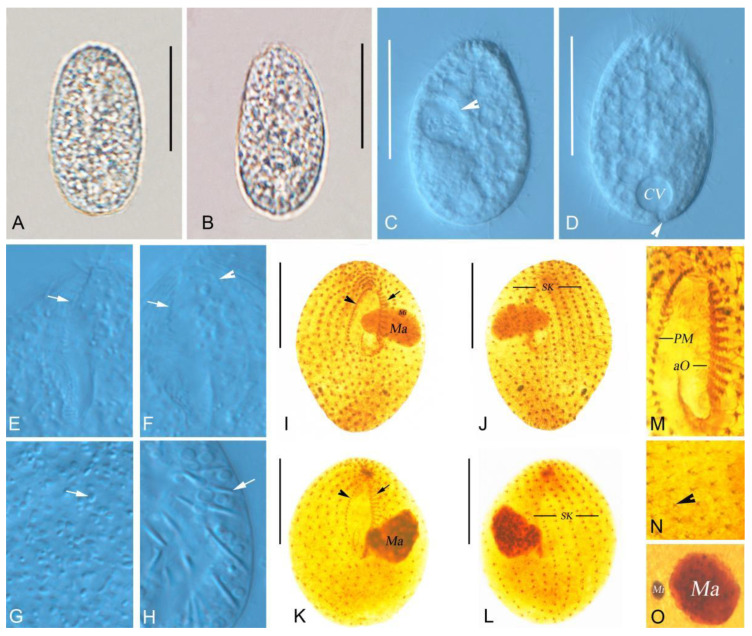
(**A**–**O**) Photomicrographs of *Bryometopus changbaishanensis* sp. n. from life (**A**–**H**) and after silver carbonate (**I**–**O**) impregnation. (**A**,**B**) Ventral views of two representative individuals. (**C**,**D**) Different individuals showing cytoplasm and contractile vacuole. Arrows in (**C**) indicate the food vacuole; in (**D**), the arrow indicates the excretory pore. (**E**–**H**) Structural details, arrows in (**E**,**F**) show oral membranelles, arrowheads show paroral membrane, arrows in (**G**) show cortical granules, arrows in (**H**) show extrusomes; (**I**–**L**) Representative individuals of *Bryometopus changbaishanensis* sp. n. after silver impregnation, arrows in (**I**,**K**) show oral membranelles, arrowheads show paroral membrane, (**J**,**L**) Dorsal views. (**M**) Detailed infraciliature of buccal area. (**N**) Somatic kineties, arrowheads showing dikinetids. (**O**) Macronucleus and micronucleus. aO, adoral organelles; CV, contractile vacuole; Ma, macronucleus; Mi, micronucleus; PM, paroral membrane; SK, somatic kineties. Scale bars: 30 μm (**A**–**D**,**I**–**L**).

The morphometric results of this study are shown in Figure 2A–F and Figure 3A–O, and Table 1.

The size in vivo was about 40–48 × 20–29 µm, and body length-to-width ratio was approximately 1.8:1. The cytoplasm is colorless, and the outline is ovoid, occasionally elongate–ovoid (Figure 2A and Figure 3A,B). The macronucleus is protruding outward and is spherical or ellipsoidal (Figure 3C), and irregularly ellipsoidal in some individuals, containing numerous nucleoli; the size in vivo is about 11–13 × 6–9 µm. The micronucleus is located near the macronucleus, enclosed by the macronucleus in some individuals (Figure 2A,C,F and Figure 3O). The contractile vacuole is situated at the posterior part of the cell (Figure 2A and Figure 3D), about 13 µm across when fully extended, with excretory pore located at the central base of the contractile vacuole (Figure 2A and Figure 3D), measuring about 2–3 × 1–2 µm. Food vacuoles 8–11 µm in diameter; multiple vacuoles ranging from very small to large, about 3–7 µm across (Figure 2A and Figure 3C,D), containing bacteria. Cortical granules ellipsoidal, irregularly arranged, about 1–2 µm in size (Figure 2E and Figure 3G). Extrusomes fusiform are partly attached obliquely to the pellicle in an irregularly crossed pattern; about 4–8 µm long at rest (Figure 2D and Figure 3H). Somatic cilia varied in length, about 5–11 µm long.

There are 11–14 somatic kineties; the buccal cavity is large and deep, occupying approximately 45–50% of the body length; the distance between the adjacent somatic kineties is about 2–3 µm. The paroral membrane is aligned alongside somatic kinety 1 and composed of about 16–26 dikinetids. (Figure 2A,B and Figure 3F,I,M,K; Table 1); the 11–15 oral membranelles are in rows (Figure 2A,B and Figure 3E,F,I,M,K; Table 1), each consisting of two longer kineties and one shorter kinety.

**Class Nassophorea Corliss, 1979.** 

**Order Colpodidiida Foissner et al., 2002.** 

**Family Colpodididae Foissner, 1995.** 

**Genus *Apocolpodidium* Foissner et al., 2002.** 

### 3.2. Apocolpodidium etoschense Foissner et al., 2002

*Apocolpodidium etoschense* Foissner et al., 2002: 492–497, Figure 114. (original description).

*Parafurgasonia zhangi* spec. nov. Fan et al., 2014: 2385–2394, Figure 1 and Figure 2. (morphological description and phylogenetic analysis based on a population from Saudi Arabia).

*Apocolpodidium etoschense* (originally described as *Parafurgasonia zhangi* spec. nov.)–2022: 173: 125,867, Figure 1. (ultrastructural study and systematic revision of the species, with reclassification from *Parafurgasonia zhangi* spec. nov.).

*Apocolpodidium etoschense* Foissner et al., 2002 was originally described by Foissner et al. (2002) [27] from Namibia and has subsequently been reported twice [27,41], with morphological variations noted among different populations. To better delimit the boundaries of this species based on morphological characters and to provide a reliable basis for identification, an updated diagnosis is provided, incorporating data from previous reports, and a new Chinese population from the Changbai Mountain region is considered in this study.

#### 3.2.1. Voucher Slides

One voucher slide (registration number WYX-2024091702) has been deposited in the Laboratory of Protozoology, Harbin Normal University, Harbin, China.

#### 3.2.2. Improved Diagnosis

The body is about 48–85 μm × 19–35 μm in vivo, and is an elongate oval in outline; contractile vacuole is at the mid-body with the excretory pore at the level of the anterior end of the postoral kineties and near the posterior end of the paroral membrane; there are 16–20 somatic kineties with about 9–11 kineties in dorsal somatic kineties; the paroral membrane is gently curved and is composed of about 13–20 dikinetids; hypostomial organelle is composed of three to five files with about three to five monokineties each.

#### 3.2.3. Description of Chinese Changbai Mountain Populations

**Figure 4:**
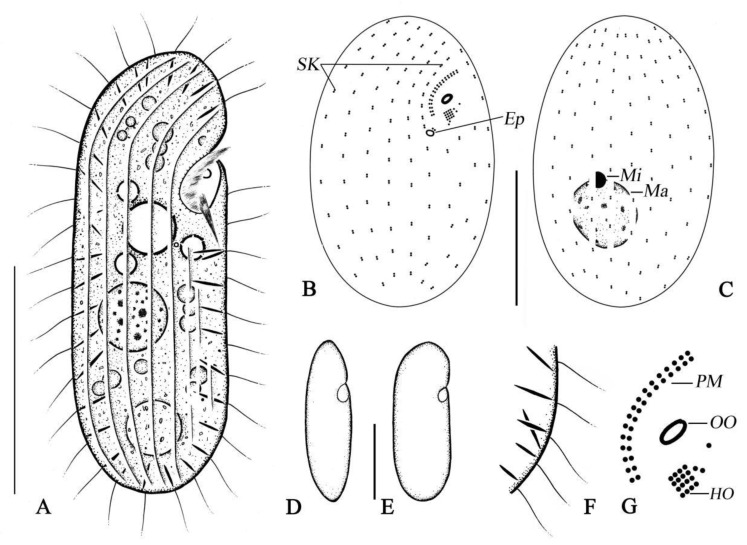
(**A**–**G**) Morphology and ciliary pattern of *Apocolpodidium etoschense* Foissner et al., 2002 from life (**A**,**D**,**E**,**F**) and after silver carbonate staining (**B**,**C**,**G**). (**A**) Typical body shape of a representative specimen. (**B**,**C**) Ventral and dorsal views of infraciliature. (**D**,**E**) Various body shapes. (**F**) Extrusomes and somatic cilia. (**G**) Oral structure after silver carbonate. Ep, excretory pore; HO, hypostomial organelle; Ma, macronucleus; Mi, micronucleus; OO, oral opening; PM, paroral membrane; SK, somatic kineties. Bar, 40 μm (**A**–**E**).

**Figure 5:**
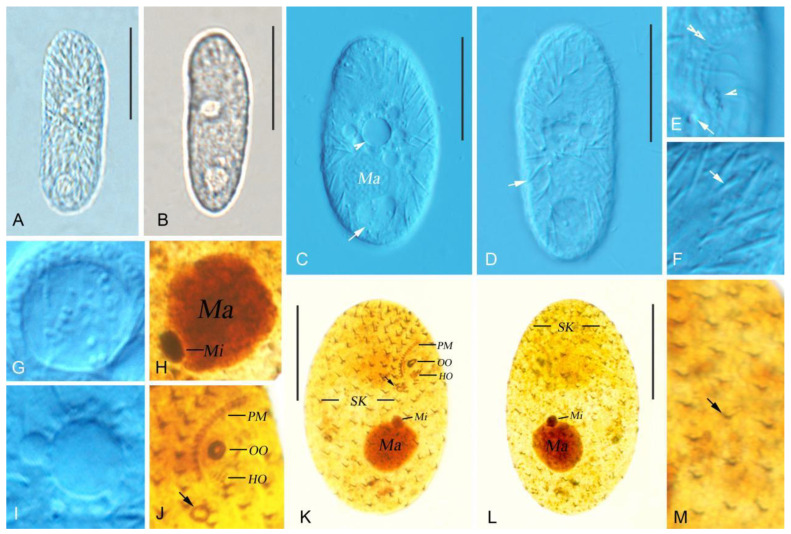
(**A**–**M**) Photomicrographs of *Apocolpodidium etoschense* Foissner et al., 2002 from life (**A**–**G**,**I**) and after silver carbonate (**H**,**J**–**M**) impregnation. (**A**,**B**) Ventral views of two representative individuals. (**C**,**D**) Different individuals. Arrows in (**C**) indicate the food vacuole; arrows in (**D**) indicate the extrusomes. (**E**) Arrows show excretory pore, arrowheads show hypostomial organelle, double arrowheads show paroral membrane. (**F**) Arrows show extrusomes. (**G**) Food vacuole. (**H**) Macronucleus and micronucleus. (**I**) Contractile vacuole. (**J**) Detailed infraciliature of buccal area, arrows show excretory pore. (**K**–**L**) Representative individuals of *Apocolpodidium etoschense* Foissner et al., 2002 after silver impregnation, arrow in (**K**) showing excretory pore. (**M**) Basical bodies of somatic kineties, arrow shows dikinetids. HO, hypostomial organelle; Ma, macronucleus; Mi, micronucleus; OO, oral opening; PM, paroral membrane; SK, somatic kineties. Scale bars: 40 μm (**A**–**D**,**K**–**L**).

The morphometric results of this study are shown in Figure 4A–G and Figure 5A–M, and Table 2.

The cell size is about 67–85 μm × 22–30 μm in vivo, and body length-to-width ratio is approximately 2.8–3:1. The cells are colorless and non-contractile. The body is an elongate oval shape when in the ventral view, the right margin slightly convex, and the left margin straight (Figure 4A and Figure 5A,B). The buccal field is subapically located, with the buccal cavity depressed (Figure 4A and Figure 5D,E). The contractile vacuole at mid-body (Figure 4A) contracts every 8 s, about 9 µm across when fully extended, and the conspicuous excretory pore located near the posterior end of the paroral membrane, about 2–3 µm across (Figure 4A,B and Figure 5E), possesses a single food vacuole, approximately 10 µm in diameter, located in the posterior part of the cell (Figure 4A and Figure 5A–D,G). Extrusomes in a resting state are about 6–10 µm long, fusiform, and partly and obliquely attached to pellicle in an irregularly crossed pattern (Figure 4A,F and Figure 5C,D,F). The macronucleus is approximately globular and in vivo is about 17 µm in diameter, usually in the middle- to posterior portion of the cell, and the micronucleus is attached to the macronucleus tightly (Figure 4A,C and Figure 5C,H,K,L). The somatic cilia are approximately 7–8 µm long. Pharyngeal rods not observed in vivo. It creeps or swims moderately fast by rotation about the main body axis.

A total of 16–20 somatic kineties, including about 9 dorsal somatic kineties, are all composed of dikinetids (Figure 4B,C and Figure 5K,L,M; Table 2). Each kinety consists of approximately 9–14 kinetids. Three or four postoral kineties are shorter, with larger intervals between adjacent kinetids.

The oral opening is elliptical, approximately 4 × 2 μm in silver-impregnated specimens, pharyngeal rods were not observed in stained preparations (Figure 4A,B,G and Figure 5J,K; Table 2); the nassulid organelle three is oriented obliquely to the main body axis, the hypostomial organelle is close underneath the oral opening, quadrate, oriented obliquely to the main body axis, and is usually composed of three or four files, each having about four basal bodies (Figure 4B,G and Figure 5D,E,J,K; Table 2); the paroral membrane is extending right and above the oral opening; gently curved and continuous with first somatic kinety, it is composed of about 16–20 dikinetids (Figure 4A,B,G and Figure 5D,E,J,K; Table 2); cytopyge inconspicuous.

#### 3.2.4. Phylogenetic Analyses

The two newly obtained SSU rDNA sequences, which have been deposited in the GenBank database, are reported here along with their respective lengths (excluding forward and reverse primers), GC content and accession numbers are reported here: *Bryometopus changbaishanensis* sp. n., 1702 bp, 43.13%, PX629844; *Apocolpodidium etoschense* Foissner et al., 2002, 1709 bp, 44.94%, PX629843; With the current new species included, the genus *Bryometopus* now comprises five species, while the genus *Apocolpodidium* still comprises one species.

**Figure 6:**
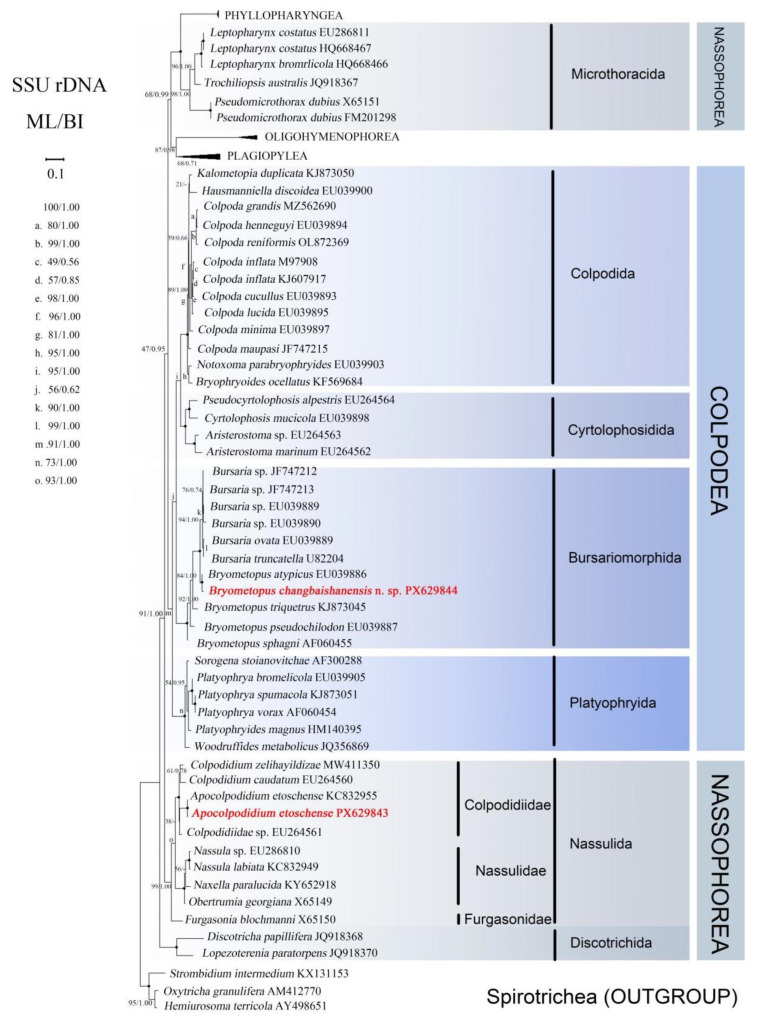
Phylogenetic tree obtained from SSU rRNA gene sequences analyses using ML and BI methods to show the positions of *Bryometopus changbaishanensis* sp. n. and *Apocolpodidium etoschense* Foissner et al., 2002 (in red and bold). The supports for nodes are indicated as follows: ML bootstraps/BI posterior probability. ‘-’ indicate mismatch in topology between Bayesian and ML trees. Fully supported (100%/1.00) clades are marked with solid circles. The scale bar corresponds to 0.1 expected substitutions per site.

The topologies of maximum likelihood (ML) and Bayesian inference (BI) trees based on the SSU rRNA gene were congruent; therefore, only the ML tree is shown with support values from both algorithms (Figure 6). The class Nassophorea is paraphyletic, being divided into three major clades with most of its lineage clustering with other various lineages. Within the clade Nassulida, which includes *A. etoschense* Foissner et al., 2002, three subclades were identified: Colpodidiidae, Nasuliidae, and Furgasonidae. The clade Nassulida, where *A. etoschense* Foissner et al., 2002 was included, was separated into three subclades, namely Colpodidiidae, Nasuliidae, and Furgasonidae. The newly obtained *A. etoschense* Foissner et al., 2002 population from Changbai Mountain and the *A. etoschense* Foissner et al., 2002 population from Saudi Arabia retrieved from the GenBank database clustered together with full support, forming a clade that then clustered with *Colpodidiidae* sp. In the ML tree, *Colpodidiidae* sp. occupied the basal position of Colpodidiida, while three species within the clade clustered together in the BI tree. Subsequently, *C. zelihayildizae* and *C. caudatum* together formed a clade that was a sister to the branch containing all three of the above species (ML/BI, 100/1.00).

The order Nassulida is more closely related to the class Colpodea, which is monophyletic, as all four orders within Colpodea are also monophyletic. Specifically, Colpodida and Cyrtolophosidida cluster together to form a clade, with Bursariomorphida representing a sister clade, while the order Platyophryida occupies a basal position within Colpodea. The newly sequenced *B. changbaishanensis* sp. n. and *B. atypicus* from GenBank form a clade with full support (ML/BI, 100/1.00), which then groups as a sister group to the genus *Bursaria* (comprising *B. ovata*, *B. truncatella*, and four unidentified populations) with full support.

## 4. Discussion

### 4.1. Comparison of Bryometopus changbaishanensis sp. n. with Congeners

Species of the genus *Bryometopus* Kahl, 1932 are primarily characterized by their size, ranging from small to large, and by a shallow vestibulum that is either parallel or oblique to the longitudinal axis of the cell. The paroral membrane extends along the right margin of the elliptical vestibular trough [19]. According to Foissner’s revision of the genus [19], the stable diagnostic features at the generic level are primarily the morphology of the vestibulum and the paroral membrane. Specifically, all *Bryometopus* species exhibit a shallow vestibulum that is either parallel or oblique to the longitudinal axis of the cell, in conjunction with a paroral membrane that extends along the right margin of the elliptical vestibular trough. These features are both conservative and reliable for generic assignment. *Bryometopus changbaishanensis* sp. n. is classified under the genus *Bryometopus* due to several distinguishing characteristics: the vestibulum is inclined towards the cell’s longitudinal axis, the paroral membrane extends into the vestibular trough, the adoral zone of organelles exhibits a slight curvature, and the ciliary pattern is notable. In contrast, several morphological traits exhibit significant variability and are therefore considered diagnostic at the species level. These traits include the following: (1) in vivo body length and width; (2) the number of somatic kineties; (3) the structure and number of adoral organelles (notably, the row count and length of kinetids may vary among species); (4) the position of the contractile vacuole (either terminal or equatorial); and (5) the body shape (elliptical versus triangular or drop-shaped). Although Foissner noted that features such as the contractile vacuole might be insufficient for generic delineation, they remain valid and are commonly used to distinguish species within the genus. *B. changbaishanensis* sp. n. conforms to the stable generic diagnosis (characterized by a shallow vestibulum and the position of the paroral membrane). However, it differs from its congeners in several species-level variable characters: its specific in vivo body dimensions (length and width), the number of somatic kineties, and the range of adoral organelles. These differences exceed the known intraspecific variability reported for other *Bryometopus* species and are consistent across all examined individuals, indicating that they represent fixed interspecific differences rather than intraspecific polymorphism.

The following nine species of Genus *Bryometopus*, which possess shallow vestibulum parallel or oblique to the longitudinal axis of the cell, should be compared in detail with the new species: *B. aypicus* [19,21]; *B. sphagni* [19,20]; *B. chlorelligerus* [19,21]; *B.viridis* [19,20]; *B. pseudochilodon* [19]; *B. edaphonus* [19,21]; *B. triquetrus* [19]; *B. balantidioides* [19]; *B. hawaiiensis* [22] (Table 3).

*B. changbaishanensis* sp. n. differs from *B. atypicus* in having a smaller body size in vivo (40–48 × 20–29 μm vs. 50–85 × 30–40 μm), fewer somatic kineties (11–14 vs. 17–30), and fewer oral membranelles (11–15 vs. 16–23) [19,21].

*B.changbaishanensis* sp. n. can be easily distinguished from *B. sphagni* by its smaller body length in vivo (40–48 μm vs. 70–150 μm), fewer micronuclei (one vs. usually >two), and fewer somatic kineties (11–14 vs. 43–60) [19,20].

*B. changbaishanensis* sp. n. differs from *B. chlorelligerus* in having a smaller body length in vivo (40–48 μm vs. 75–95 μm), fewer somatic kineties (11–14 vs. 40), fewer micronuclei (one vs. two), and fewer oral membranelles (11–15 vs. 25) [19,21].

*B. changbaishanensis* sp. n. can be distinguished from *B. viridis* primarily by its smaller body length in vivo (40–48 μm vs. 70–115 μm), fewer somatic kineties (11–14 vs. 70–80), and fewer oral membranelles (11–15 vs. 45–60) [19,20].

*B. changbaishanensis* sp. n. can be morphologically distinguished from *B. pseudochilodon* by having fewer micronuclei (one vs. two to nine), fewer somatic kineties (11–14 vs. 34–42), and fewer oral membranelles (11–15 vs. 22–30) [19].

*B. changbaishanensis* sp. n. differs from *B. edaphonus* in having fewer somatic kineties (11–14 vs. 16–20), fewer oral membranelles (11–15 vs. 25), and fewer dikinetids in the paroral membrane (16–26 vs. 37) [19,21].

*B. changbaishanensis* sp. n. most conspicuously differs from *B. triquetrus* in body shape (ovoid vs. triangular). It can be further distinguished by having fewer somatic kineties (11–14 vs. 16–20), fewer oral membranelles (11–15 vs. 16–27), and fewer dikinetids in the paroral membrane (16–26 vs. 35–47) [19].

*B. changbaishanensis* sp. n. can be distinguished from *B. balantidioides* by its smaller body length in vivo (40–48 μm vs. 50–70 μm), fewer somatic kineties (11–14 vs. 15–16), and fewer oral membranelles (11–15 vs. 25–26) [19].

*B. changbaishanensis* sp. n. can be distinguished from *B. hawaiiensis* by its smaller body length in vivo (40–48 μm vs. 50–70 μm), fewer somatic kineties (11–14 vs. 25–30), and a reduced number of adoral membranelles (11–15 vs. 31–42) [22].

Pairwise genetic distances based on SSU rRNA gene sequences were calculated using MEGA 12 [40] with the Kimura 2-parameter model. The newly identified *Bryometopus changbaishanensis* sp. n. exhibited a genetic distance of 0.008 from its phylogenetically closest congener, *B. atypicus*. Comparisons with other members of the genus *Bryometopus* revealed interspecific distances ranging from 0.05 to 0.07 (Table 4). According to previous studies on ciliate systematics, the intraspecific SSU rRNA genetic distance threshold for Mobilid ciliates typically ranges from 0.000 to 0.005, while the interspecific (congeneric) threshold ranges from 0.005 to 0.150 [42]. Similar thresholds have also been reported in the genus *Trichodina* [43]. The genetic distances observed between *B. changbaishanensis* sp. n. and its congeners in this study all exceed the intraspecific threshold and fall within the typical interspecific range. In terms of morphological characteristics, *B. changbaishanensis* sp. n. differs from other species within the genus in its in vivo body length and width, the number of somatic kineties, and the range of adoral organelles. The combination of the molecular evidence presented above with these morphological characteristics strongly supports the recognition of this isolate as a new species.

### 4.2. Identification of the Chinese Changbai Mountain Population of Apocolpodidium etoschense Foissner et al., 2002 and Comparison with Its Previously Reported Conspecific Populations

Since the overall morphological characteristics of *Apocolpodidium etoschense* Foissner et al., 2002 have been described in detail previously, with three populations reported worldwide from Namibia in Africa, Austria in Europe, Saudi Arabia in Asia [27,41], and the population described here from the Changbai Mountains, China. This is the first report of this species from China. The previously described populations measured 48–63 × 25–35 μm in vivo, with 16–19 somatic kineties and 13–19 dikinetids in the paroral membrane. The Changbai Mountain population corresponds well with these three characteristics. Further details are provided in (Table 5).

### 4.3. Phylogenetic Analyses

To date, the genus *Bryometopus* comprises a total of ten species: *B. atypicus*, *B. sphagni*, *B. chlorelligerus*, *B. viridis*, *B. pseudochilodon*, *B. edaphonus*, *B. triquetrus*, *B. balantidioides*, *B. hawaiiensis*, and *B. changbaishanensis* sp. n. Among these, genetic sequence data are currently unavailable for *B. chlorelligerus*, *B. viridis*, *B. edaphonus*, and *B. balantidioides*. Consequently, the phylogenetic tree constructed in this study includes sequence data from only the remaining five species within the genus *Bryometopus*.

In the SSU rRNA gene phylogeny, the genus *Bryometopus* is paraphyletic and shows a close relationship with the genus *Bursaria*, which is consistent with previous studies [44,45]. The sequence of the new species, *Bryometopus changbaishanensis* sp. n., clusters within the *Bryometopus* clade with high support values (Figure 6). This clustering is highly congruent with the shared morphological characteristics defining the genus *Bryometopus*: (1) with a shallow vestibulum that is either parallel or oblique to the longitudinal axis of the cell; (2) the paroral membrane extending along the right margin of the elliptical vestibular trough [19]. *B. changbaishanensis* sp. n. and *B. atypicus* (Accession No. EU039886) cluster together with full support (100% ML/1.00 BI). This close relationship is further corroborated by morphological evidence (e.g., “the shared characteristics of these two species”). The two species differ by 11 nucleotides, and considering their morphological distinctions, this supports the establishment of the former as a new species while still retaining *B. changbaishanensis* sp. n. within the genus *Bryometopus*.

It is noteworthy that the *Bryometopus* species, which form a sister clade to the genus *Bursaria,* comprise only two species. Therefore, more integrative studies and comprehensive sampling are required to reliably elucidate the evolutionary relationships within Bursariomorphida.

Our molecular phylogenies based on SSU rRNA genes indicate that the class Nassophorea is nonmonophyletic; Microthoracida is distant from the Nassulida and Discotrichida, and Colpodidiidae is monophyletic and clusters within Nassulida. These findings are consistent with those obtained in previous studies [41,46,47]. The sequence of *Apocolpodidium etoschense* (PX629843), obtained in this study, is consistent with the one available on NCBI, *A. etoschense* (KC832955) and both branches together, with low support values as the sister clade to *Colpodidiidae* sp. (EU264561). Subsequently, this entire clade is clustered together with *Colpodidium zelihayildizae* and *Colpodidium caudatum*, consistent with the topology reported in previous studies [26].

Morphologically, these two genera can be clearly distinguished by the characteristics of their buccal apparatus: the genus *Colpodidium* possesses a horn-shaped or pocket-shaped buccal cavity directed anteriorly or to the right side, whereas the genus *Apocolpodium* possesses a keyhole-shaped buccal cavity that is either small and slightly concave or large and rather deep [27]. Given the lack of morphological and molecular data for many species, it is recommended to follow the suggestions of recent relevant studies and adopt an integrative taxonomy approach for further research, including the description and redescription of species [48,49].

## Figures and Tables

**Table 1:** Morphometric characterization of *Bryometopus changbaishanensis* sp. n. based on silver carbonate specimens.

Character	Min	Max	Mean	Med	SD	CV	n
Body length, μm	46	71	60.3	62	7.7	12.7	15
Body width, μm	34	59	42.4	42	5.5	13.0	15
Macronucleus, number	1	1	1.0	1	0.0	0.0	15
Macronucleus length, μm	14	25	19.6	20	3.7	19.0	15
Macronucleus width, μm	6	17	12.2	12	3.0	24.8	15
Micronucleus, number	1	1	1.0	1	0.0	0.0	15
Micronucleus length, μm	3	4	3.5	4	0.5	14.6	15
Micronucleus width, μm	2	3	2.6	3	0.5	19.5	15
Somatic kineties, number	11	14	12.9	13	1.0	7.7	14
Adoral organelles, number	11	15	13.6	14	1.4	10.3	15
Adoral dikinetids, number	16	26	21.1	22.0	3.2	15.2	14

**Table 2:** Morphometric characterization of *Apocolpodidium etoschense* Foissner et al., 2002 from the Changbai Mountain region, China, based on silver carbonate specimens.

Character	Min	Max	Mean	Med	SD	CV	n
Body length, μm	61	87	74.6	76.0	7.8	10.5	14
Body width, μm	31	53	44.4	47.0	7.4	16.7	14
Macronucleus, number	1	1	1.0	1.0	0.0	0.0	14
Macronucleus diameter, μm	12	21	17.3	17.0	2.6	15.3	14
Micronucleus, number	1	1	1.0	1.0	0.0	0.0	14
Micronucleus diameter, μm	3	5	4.0	4.0	0.8	19.6	14
Oral opening length, μm	3	4	3.3	3.0	0.5	14.3	14
Oral opening width, μm	2	3	2.2	2.0	0.4	19.2	14
Anterior body end to oral opening distance, μm	15	27	21.8	23.0	4.0	18.2	14
Anterior body end to excretory pore distance, μm	22	35	30.3	32.0	3.9	12.9	14
Anterior body end to macronucleus distance, μm	30	46	39.9	40.0	5.0	12.6	14
Somatic kineties, number	16	20	17.1	17.0	1.1	6.7	14
Paroral membrane, number	16	20	17.9	18.0	1.0	5.6	14

**Table 3:** Morphological comparison of *Bryometopus changbaishanensis* sp. n. with their most related congeners.

Species	Size In Vivo, μm	Somatic Kineties, Number	Adoral Organelles, Number	Adoral Dikinetids, Number	Reference
*B.changbaishanensis* sp. n.	40–48 × 20–29	11–14	11–15	16–26	Present
*B. atypicus*	50–85 × 30–40	17–23	17–23	23–30	[19,21]
*B. sphagni*	70–150 × 40–80	43–60	40–54	Absent	[19,20]
*B. chlorelligerus*	75–95	40	25	Absent	[19,21]
*B.viridis*	70–115 × 37–70	70–80	45–60	Absent	[19,20]
*B. pseudochilodon*	40–80	34–42	22–30	Absent	[19]
*B. edaphonus*	40–60	16–20	25	37	[19,21]
*B. triquetrus*	45–55 × 25–35	16–20	16–27	35–47	[19]
*B. balantidioides*	50–70 × 25–35	15–16	25–26	26–30	[19]
*B. hawaiiensis*	50–70 × 35–45	25–30	31–42	24–36	[22]

**Table 4:** Pairwise genetic distances based on SSU rRNA gene sequences between *Bryometopus changbaishanensis* sp. nov. and its congeners.

Species Comparison	SSU rRNA Genetic Distance
*B.changbaishanensis* sp. n. vs. *B. atypicus*	0.008
*B.changbaishanensis* sp. n. vs. *B. triquetrus*	0.057
*B.changbaishanensis* sp. n. vs. *B. pseudochilodon*	0.060
*B.changbaishanensis* sp. n. vs. *B. sphagni*	0.067

**Table 5:** Morphological comparison of *Apocolpodidium etoschense* Foissner et al., 2002 with its previously reported conspecific populations.

	Isolated	Size In Vivo, μm	Macronucleus Size, μm	Micronucleus Diameter, μm	Somatic Kineties, Number	Paroral Membrane, Number	Reference
*Apocolpodidium etoschense* Foissner et al., 2002	China	67–85 × 22–30	12–21(diameter)	3–5	16–20	16–20	Present
	Saudi Arabia	48–63 × 19–31	8–15(diameter)	2–4	16–17	13–17	[27,41]
	Namibia	55–75 × 25–35	9–11 × 6–10	3	16–18	13–17	[27]
	Austria	Absent	8–14 (diameter)	Absent	18–19	16–19	

## Data Availability

The original contributions presented in this study are included in the article. Further inquiries can be directed to the corresponding author.
